# Type-1 Cannabinoid Receptor Promiscuous Coupling: Computational Insights into Receptor-G Protein Interaction Dynamics

**DOI:** 10.3390/ijms262411905

**Published:** 2025-12-10

**Authors:** Alessandro Berghella, Tomasz Maciej Stepniewski, Annalaura Sabatucci, Marta Lopez-Balastegui, Krzysztof Nowicki, Beatrice Dufrusine, Jana Selent, Enrico Dainese

**Affiliations:** 1 Department of Bioscience and Technology for Food, Agriculture and Environment, University of Teramo, 64100 Teramo, Italy; aberghella@unite.it (A.B.); alsabatucci@unite.it (A.S.);; 2 Research Program on Biomedical Informatics, Hospital del Mar Research Institute, 08003 Barcelona, Spain; tm.stepniewski@gmail.com (T.M.S.);; 3 InterAx Biotech AG, 8952 Schieren, Switzerland; 4 Faculty of Chemistry, Warsaw University of Technology, Noakowskiego 3, 00-664 Warsaw, Poland

**Keywords:** CB1 receptor, endocannabinoid system, molecular dynamics, GPCR, Gs protein

## Abstract

Cannabinoid receptor (CB1), a G protein coupled receptor (GPCR), is a known pharmacological target in several diseases and modulates key physiological processes through Gi protein-mediated signaling. However, recent evidence suggests that CB1 can also activate other G proteins, including the stimulatory Gs protein, a phenomenon with unclear structural determinants. Here, we use a computational approach to elucidate the structural basis of the CB1-Gs interaction. Protein–protein docking and extensive molecular dynamics simulations yield a model for the CB1-Gs complex that agrees well with both existing experimental data and available GPCR-Gs structures, supporting its validity. This work provides new insights into the structural basis of CB1’s ability to couple with different G-proteins. The model provides a basis for future studies dissecting the functional consequences of CB1-Gs signaling and the development of improved therapeutics targeting the CB1 receptor and the wider endocannabinoid system.

## 1. Introduction

Type-1 cannabinoid receptor (CB1), together with Type-2 cannabinoid receptor (CB2), is a pivotal component of the endocannabinoid system (ECS) and assumes a central role in various pathophysiological processes like regulation of appetite, pain perception, anxiety, and neurodegeneration, as extensively reviewed [1,2]. As a G protein-coupled receptor (GPCR), CB1 is located within the cell membrane primarily in central nervous system (CNS) cells, and its function is highly dependent on the membrane environment [3]. CB1 is known to recognize a diverse range of orthosteric and allosteric modulators [4,5]. Binding of such molecules to CB1 can trigger a range of downstream signaling events, including modulation of adenylate cyclase (AC), regulation of ion channels, induction of receptor-mediated Ca^2+^ fluxes, stimulation of phospholipases, and activation of mitogen-activated protein kinase (MAPK) pathways [6,7,8]. CB1 signaling primarily relies on its interaction with heterotrimeric G proteins consisting of α, β, and γ subunits. Critically, the identity of the Gα subunit subtype serves as a major determinant of the downstream intracellular signaling outcomes [9]. Although CB1 is predominantly characterized by its coupling to Gi/o proteins [6] resulting in adenylate cyclase inhibition and decreased cAMP levels, an expanding body of evidence indicates that CB1 agonists can additionally drive coupling to Gs and Gq/11 proteins [10,11,12,13,14,15,16]. X-ray and Cryo-EM studies have provided valuable insights into the structural determinants for CB1-Gi protein complex formation [17,18,19,20] and into the dynamics of CB1 activation [17,19,21,22], but the structure of CB1 in complex with the Gs protein has not yet been resolved, and experimental data on the relationship between Gs and CB1 remains sparse. Mutational analyses show that ICL2 contributes selectively to Gs coupling, while having little or no involvement in Gi recognition [23,24]. This is in line with structural data which shows relatively scarce interactions between ICL2 and the Gαi subunit in the CB1-Gi complex (PDB:6N4B). Gaining insight into the structure of the CB1-Gαs complex could not only expand our knowledge on CB1 functionality but also help better understand common features which drive G protein selectivity among GPCRs. Finally, a comparative analysis of Gαs- and Gαi-bound CB1 complexes could yield structural insights that guide the design of agonists capable of selectively promoting Gi or Gs recruitment. Such molecules could serve as molecular probes to study the involvement of Gi and Gs recruitment in physiological processes, as well as potential starting points for the development of novel CB1 targeting drugs. In this work, we address this knowledge gap by using a combination of bioinformatic approaches to model a putative CB1–Gs complex. Our results offer key insights into the dynamic interplay between the receptor and the G protein and highlight structural features that may underlie G-protein selectivity at CB1.

## 2. Results

### 2.1. CB1-Gαs Complex In Silico Modelling

**Figure 1:**
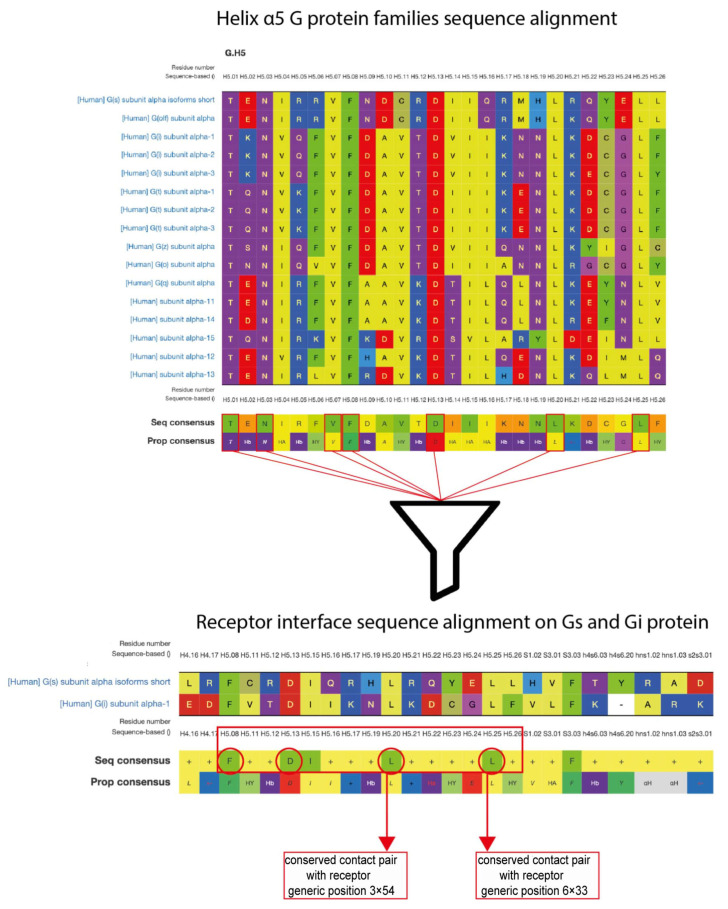
Workflow outlining the selection of structural restraints for Gs–CB1 complex docking.

To establish an initial structural model for the CB1–Gαs complex, we employed protein–protein docking with a minimal set of restraints, allowing the Gαs subunit to explore multiple conformations relative to the receptor. To select a set of restraints for the docking process we have elaborated a workflow, based on available structural GPCR data (Figure 1). Using GPCRdb [25], we first identified seven residues within the α5 helix (H5) that are fully conserved across human Gα subfamilies (C H5.01, H5.03, H5.07, H5.08, H5.13, H5.20, and H5.25). Because our goal was to investigate a potentially promiscuous interaction between Gαi- and Gαs-coupled orientations, we next examined which of these conserved H5 residues are shared between Gαi and Gαs interfaces in available GPCR–G protein complexes. This analysis identified four shared positions: H5.08, H5.13, H5.20, and H5.25. We then evaluated whether these four positions form conserved contacts with GPCRs across available structures. Structural inspection of GPCR–G protein complexes, including CB1–Gαi (PDB: 6N4B, 6KPG, 7WV9, 8GHV), revealed that only H5.20 and H5.25 consistently establish receptor contacts compatible with both Gi and Gs engagement, in agreement with previous reports [26,27]. Therefore, we selected H5.20–3.54 and H5.25–6.33 (Gαs L388–CB1 I218 and Gαs L393–CB1 L341) as docking restraints and excluded positions H5.08 and H5.13 due to their limited structural involvement in GPCR recognition.

**Figure 2:**
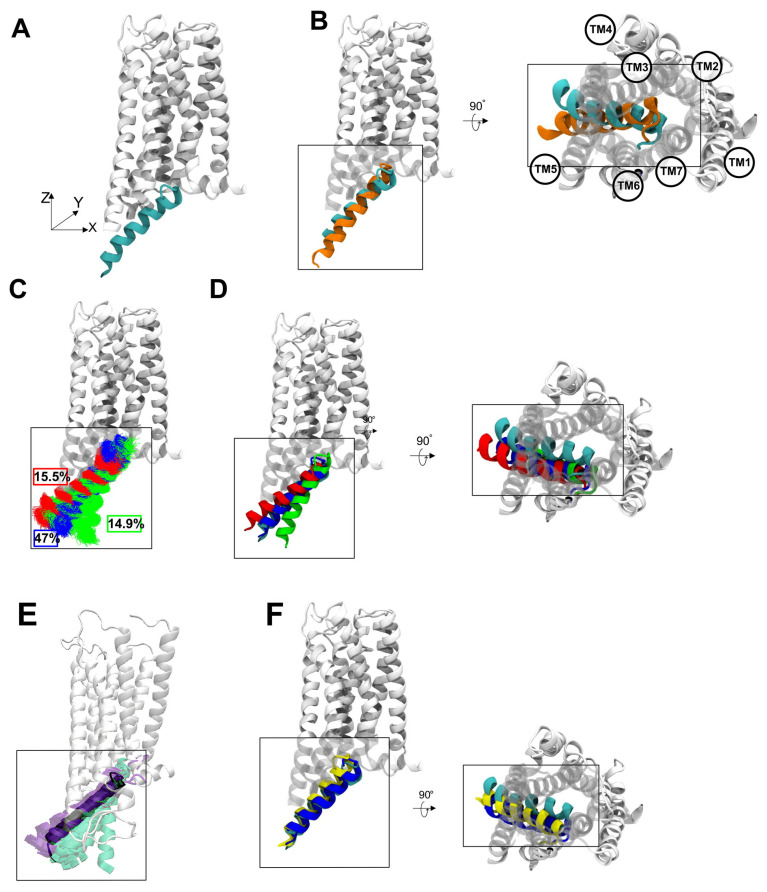
Structural analysis of Gαs α5 helix orientation and comparison with other complexes. (**A**) α5 helix orientation of the docked CB1-Gαs subunit complex (cyan). (**B**) Comparison of the α5 helix position of the docked complex vs. the α5 helix of Gαi subunit (orange). (**C**) Clustering of the position of the Gαs helix observed during 12 independent MD runs (blue cluster—47% of frames—3525 frames, red cluster—15.5% of frames—1162 frames, green cluster—14.9% of frames—1117 frames). (**D**) Most representative structure of each cluster, compared with the original docking conformation of Gαs subunit. (**E**) Orientation of the Gαs α5 helix after MD stabilization (in black tube representation) compared with Gαs (purple) and Gαi (aquamarine)-bound GPCRs structures. (**F**) Comparison of a representative structure of the most populated cluster of the Gαs subunit (blue) with the initial docking pose (cyan) and the position of the α5 helix derived from the structure of the β2-Adrenergic receptor [PDB code: 3SN6] in yellow.

The orientation of the C-terminal fragment of Gαs H5 in the resulting docking complex (Figure 2A) resembled general characteristics of the Gαi H5, when comparing the model with the CryoEM Gi/CB1 complex (Figure 2B). However, in the XZ-plane the Gαs H5 is strongly shifted towards the receptor. Furthermore, the C-terminal fragment of the helix is oriented towards TM4 and ICL2, whereas the Gαi H5 packs against TM5. Interestingly, more pronounced contacts of Gαs with ICL2 are in line with experimental results [24], which highlight this region as selectively affecting Gαs recruitment.

### 2.2. ICL3 Modelling

Following protein docking, intracellular loop 3 (ICL3) was modeled, as this region was missing from the initial structure. During this step, we excluded conformations that formed preferential interactions with either the G protein or the membrane, as well as overly folded loop arrangements. Our aim was to select a neutral, unobstructed conformation that preserves loop flexibility and avoids introducing artificial constraints, thereby ensuring a physiologically relevant representation of the CB1–Gs protein complex.

### 2.3. Molecular Dynamics Simulations Analysis

To evaluate the stability of the modeled complex, we performed extensive molecular dynamics (MD) simulations consisting of 12 independent 1-µs runs. Our rationale behind employing such an extensive sampling protocol was to allow the G protein–receptor interface to relax and adopt a physiologically relevant conformation. To ensure that our analysis reflects the dynamics of a fully engaged CB1-Gαi complex rather than early equilibration artifacts, we excluded the first half of each trajectory from subsequent analyses.

Upon analyzing the motions of the α5 helix, we could discern three predominant clusters that collectively account for nearly 80% of the simulation frames (Figure 2C). Examination of the representative conformation from each cluster in the XZ-plane (Figure 2D) shows that while the C-terminal segment of the α5 helix remains relatively fixed, the N-terminal segment exhibits substantial conformational variability. Notably, despite this variability, the most populated cluster retains an XZ-plane orientation consistent with the original docking pose. In contrast, projections onto the XY-plane reveal a consistent translation of the α5 helix toward TM5 and TM6 across all clusters, resulting in increased contacts with these transmembrane segments. Although the magnitude and uniformity of this shift may appear unexpected, it is in fact consistent with experimental observations: in GPCR–G protein complexes, the Gαs α5 helix typically forms more extensive interactions with TM5 and TM6 than does Gαi [27].

To evaluate how closely the α5 helix conformations from our most populated cluster resemble those observed in other GPCRs, we performed a comparative analysis using 81 high-resolution GPCR–G protein complexes (28 Gs and 53 Gi). We first compared the overall orientation of the α5 helices (Figure 2E). Strikingly, the α5 helix orientation in our model aligns closely with that of Gs-bound complexes, indicating strong structural compatibility. For a more detailed comparison, we selected the prototypical Gs-coupled receptor—the β_2_-adrenergic receptor (PDB: 3SN6). Upon aligning the receptor structures, we observed an unexpected degree of similarity: in both complexes the α5 helix packs tightly against the intracellular interface of TM5 and TM6 and adopts a comparable XZ-plane orientation (Figure 2F). Recovering such a conformation spontaneously in unbiased simulations strongly supports the validity of our modeled CB1–Gαs complex.

**Figure 3:**
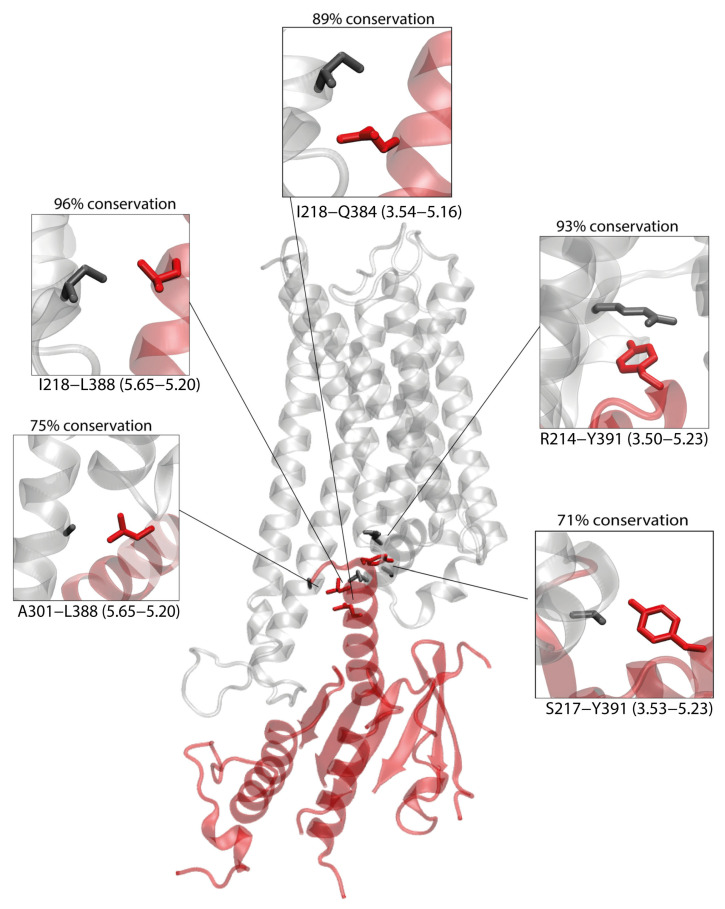
CB1-Gαs complex obtained by protein–protein docking. Five contacts present within the complex, which are conserved within GPCR/Gs complexes (see Methods) are depicted, with CB1 residues depicted in black and Gαs residues depicted in red. The conservation of each contact among Gs complexes is calculated as percentage of conservation.

We next examined whether the putative CB1–Gs interface reflects interaction patterns found in experimentally resolved GPCR–Gs structures. Using the 28 high-resolution human class A GPCR–Gs complexes available in GPCRdb (see Methods), we analyzed the conserved “General class A GPCR–Gs contact pairs” matrix. Within this matrix, six contact pairs are conserved in at least 77% of the structures (Supplementary Figure S1). Of these, five are preserved in our model—3.50-H5.23, 3.53-H5.23, 3.54-H5.16, 3.54-H5.20, and 5.65-H5.20—further supporting the structural plausibility of our CB1–Gαs complex (Figure 3).

### 2.4. The Role of the ICL2 in the CB1-Gαs Complex

**Figure 4:**
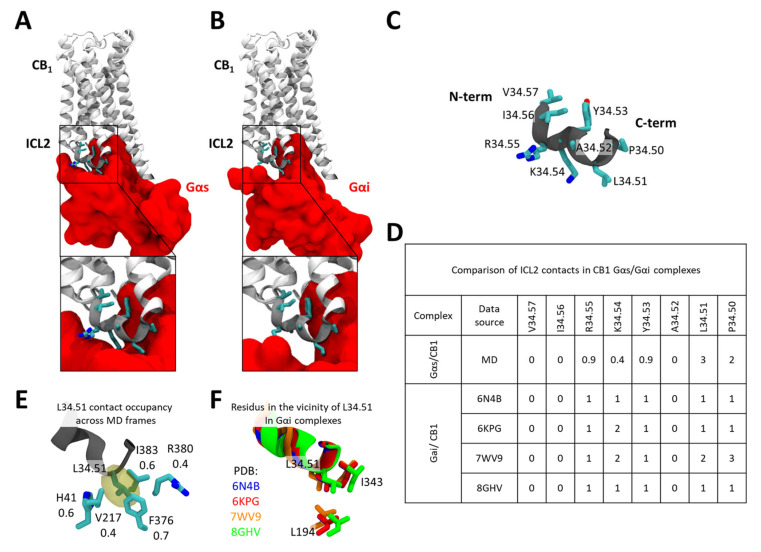
Comparison of ICL2 interaction with Gαs and Gαi bound CB1. (**A**) The interface between CB1 ICL2 and Gsα. (**B**) The interface between CB1 ICL2 and Gαi. (**C**) Sequence composition of the CB1 ICL2. (**D**) Comparison of CB1 ICL2/Gα contacts between the CB1-Gαs model (as studied in MDs) and CB1-Gαi structures available in the PDB database. (**E**) Stability of contacts formed by L34.51 and residues within the Gαs. (**F**) Contacts formed by L34.51 and residues within the Gαi in CB1 complexes.

As mentioned before, ICL2 has been highlighted to have a crucial impact on Gαs recruitment by CB1. Consistent with this, our docking results already indicate that ICL2 forms tighter interactions with Gαs than with Gαi (Figure 4A vs. Figure 4B). A more detailed comparative analysis of ICL2 contacts—using accumulated MD frames for the Gαs complex and available static structures for Gαi—reveals a markedly higher number of interactions with Gαs, particularly within the C-terminal segment of ICL2 (Figure 4C,D). Among these, residue L34.51 exhibits the highest number of contacts with the Gαs α-subunit, suggesting that it may serve as a primary anchoring point for Gαs. This interpretation is fully consistent with experimental findings showing that mutation of this site strongly impairs Gαs recruitment [24].

A more detailed analysis of the contacts formed by L34.51 reveals that, within MD simulations, it nests within a hydrophobic pocket formed by Gαs residues F376^H5.15^, V217^S3.01^, I383^H5.15^, and the hydrophobic portions of R380^H5.12^ and H41^S1.02^ (Figure 4E). Such a tight fit between L34.51 and the Gαs provides a mechanistic explanation for why substitutions to smaller side chains (e.g., V, A, or I) markedly reduce Gαs coupling efficiency, as reported by Chen et al. [24], whereas introducing a bulkier residue (e.g., phenylalanine) does not. In contrast, in available CB1–Gαi complexes, L34.51 forms far fewer contacts (Figure 4F), which may explain why mutations at this position have a comparatively lesser effect on Gi recruitment.

### 2.5. ICL3 Dynamics and Interactions During the CB1–Gαs Simulations

**Figure 5:**
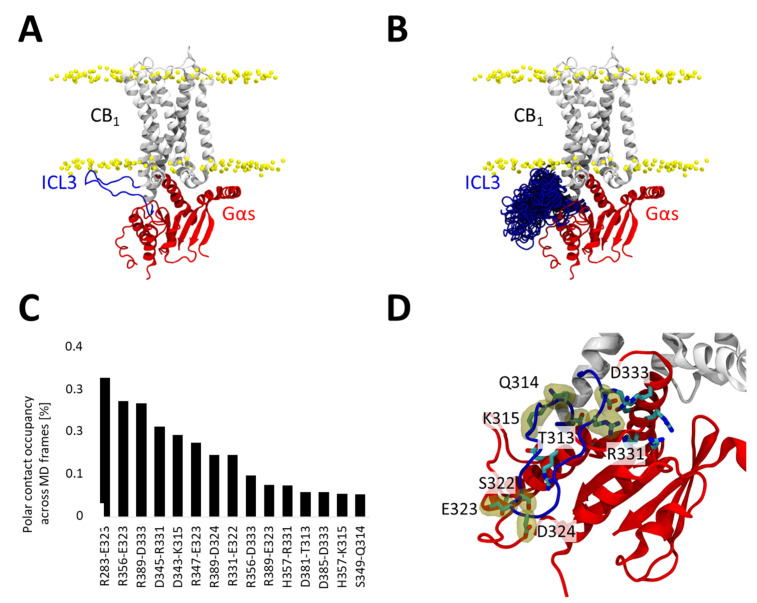
Contribution of ICL3 to the CB1–Gαs interaction. (**A**) Initial CB1-Gαs used for MD simulations. ICL3 (residues 312 to 336) are colored in blue and Gαs in red. (**B**) Conformational ensemble explored by ICL3 during MD simulations, we depict the ICL3 conformation once every 100 ns (60 snapshots in total, 10 snapshots per replicate). (**C**) Polar interactions formed between ICL3 residues and the Gαs subunit. ICL3 residues are listed at the bottom of each label. We depict only interactions occurring during at least 0.05 of the trajectories. (**D**) Structural location of residues forming polar interactions in the ICL3 and the Gαs subunit. ICL3 residues are labelled and highlighted with a yellow surface.

Because the model incorporated ICL3 in an extended conformation—introduced de novo after docking (Figure 5A)—we also sought to evaluate the potential interactions this flexible region could form with the G protein. To this end, we analyzed both its conformational behavior and its contribution to the CB1–Gαs interface over the accumulated simulations (Figure 5B). As expected for an intrinsically flexible loop, it does not converge to a single stable conformation (Figure 5B). Residue–residue hydrogen-bond occupancies between ICL3 and Gαs were quantified across the full trajectories using VMD-based analyses (Figure 5C). The complete contact map and the 15 residue pairs with the highest hydrogen-bond occupancies are shown in Figure 5C,D. The calculated contacts suggest that despite not converging towards a single conformation, ICL3 consistently forms a transient interaction network (Figure 5C,D) with the Gαs which may contribute to stabilizing the complex.

### 2.6. Key Residues Involved in the Interaction CB1-Gαs Complex

**Figure 6:**
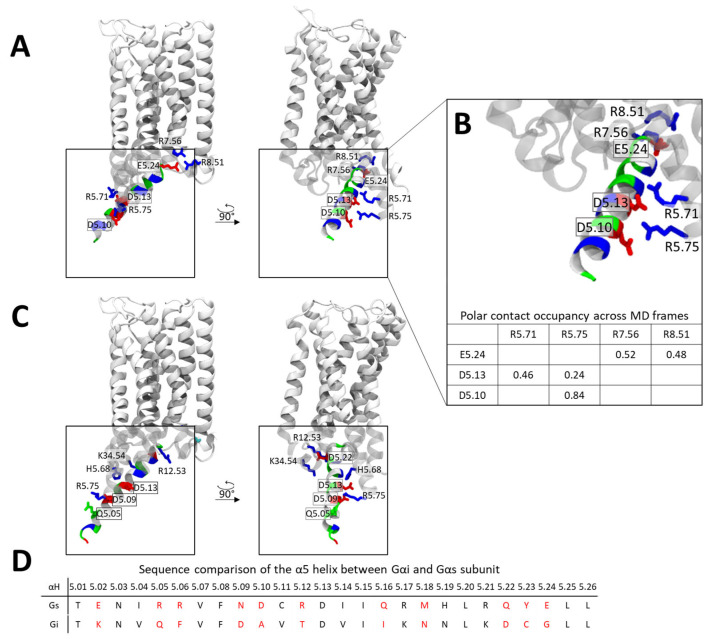
Contact patterns of the α5 helix within the CB1-Gαi and CB1-Gαs complexes. (**A**) Representative conformation of the α5 Gαs helix in complex with CB1 extracted from MD simulations, residues are colored based on charge (red—negative, blue—positive, green—polar). (**B**) Polar contacts formed by the Gαi α5 helix with CB1, residues are colored based on charge (red—negative, blue—positive, green—polar) stability of polar contacts is summarized below in a table. (**C**) Conformation of the a5 Gαi helix in complex with CB1 [PDB code: 6N4B], residues are colored based on charge (red—negative, blue—positive, green—polar). (**D**) Sequence comparison of the α5 helix between Gαi and Gαs. Positions with non-conserved residue types are indicated in red.

Given the strikingly different conformations adopted by the Gαi and Gαs α5 helices within the CB1 receptor, we sought to identify the molecular features that drive these distinct binding modes. Analysis of the Gαs α5 helix contacts with CB1 reveals that E5.24, located at the C-terminal end of the helix, is positioned near two positively charged residues at the intracellular end of TM7 and H8 (R7.56 and R8.51) (Figure 6A). Contact stability analysis indicates that these polar interactions are highly persistent, occurring in ~52% (R7.56) and ~48% (R8.51) of the studied simulation frames. In addition, negatively charged residues in the central region of the α5 helix (D5.10 and D5.13) form polar interactions with arginine residues in TM5 (R5.71 and R5.75) (Figure 6B). Together, these interactions effectively “lock” the α5 helix in place through a zipper-like arrangement involving three negatively charged α5 residues engaging three clusters of positive charges on CB1.

In contrast, the Gαi α5 helix exhibits a distinct interaction pattern. Here, a C-terminal negatively charged residue (D5.22) forms polar contacts with a cluster of positively charged residues in ICL1 and ICL2 (Figure 6C). Additional stabilization is provided by interactions between D5.13 and H5.68, and between D5.09 and Q5.05 with R5.75. When comparing the two complexes, it becomes clear that each helix relies on a different set of acidic residues for stabilization: D5.10, D5.13, and E5.24 for Gαs, and D5.08, D5.13, and D5.22 for Gαi. In both cases, acidic residues in the central portion of the helix (D5.10 and D5.13 in Gαs; D5.08 and D5.13 in Gαi) interact with polar or charged residues in TM5, while C-terminal acidic residues further stabilize the helix and dictate its orientation in the XY-plane.

Importantly, Gi lacks a negatively charged residue at position 5.24 and instead contains an aspartate at position 5.22 (Figure 6D). Due to geometric constraints, D5.22 cannot form stabilizing interactions with the TM7/H8 cluster, causing the α5 helix to rotate toward the ICL1/ICL2 region. This structural difference likely underlies the divergent α5 helix engagement modes observed for Gαs and Gαi.

### 2.7. Dynamics of TM5 and TM6 in the CB1-Gαs Complex

Finally, we examined conformational differences in the CB1 receptor when bound to either Gαi or Gαs. We focused on regions that commonly exhibit the greatest structural rearrangements during G-protein coupling—namely, the intracellular portions of TM5 and TM6. Clustering these regions and visualizing the representative structures of the most populated clusters revealed that, in the Gαs-bound complex, both helices undergo a noticeably larger displacement compared to the Gαi-bound structure. This observation is consistent with structural data showing that Gs-coupled GPCRs typically display a more pronounced TM6 outward movement than Gi- or Gq-coupled receptors [28].

**Figure 7:**
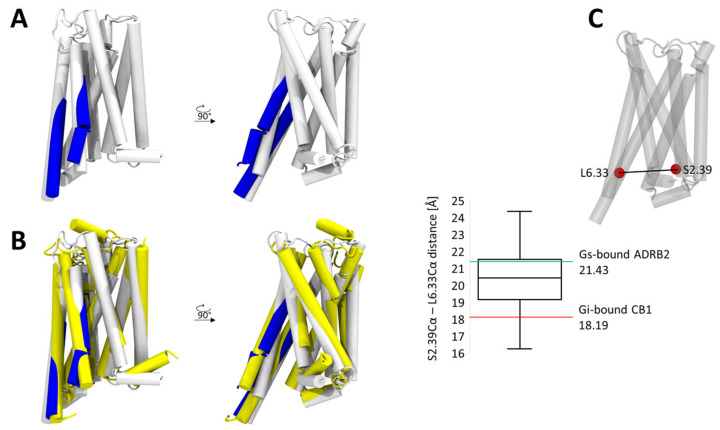
Dynamics of TM5 and TM6 in the CB1-Gαs complex. (**A**) Results of clustering of TM5 and TM6 positions, representative structures of the most populated cluster are depicted in blue and aligned with the structure of the Gi bound CB1. (**B**) Comparison of the positions of TM5 and TM6 extracted from the clustering analysis, with that of the Gi bound CB1 (white) and Gαs-bound β2-Adrenergic receptor (yellow). (**C**) Box plot of the distance between TM2 and TM6 (residues 6.33 and 2.39) observed in the simulations. The distance corresponds more to a Gαs-bound β2-Adrenergic receptor structure rather than to a Gi bound one.

Analysis of the TM2–TM6 distance across simulation frames further indicates that the CB1–Gαs complex predominantly samples conformations with a wider separation than those observed in the CB1–Gαi structure (Figure 7A). Interestingly, although the TM6 opening occasionally reaches values similar to those seen in the Gαs-coupled β_2_-adrenergic receptor (a prototypical Gs-preferring GPCR), the average displacement remains smaller than in that complex (Figure 7B,C). This trend aligns with structural data demonstrating that GPCRs with primary Gs coupling tend to exhibit larger TM6 openings when bound to Gs [29], whereas receptors that preferentially couple to Gi display comparatively smaller TM6 displacements when interacting with Gs.

## 3. Discussion

Recent advances in elucidating GPCR selectivity demonstrated widespread promiscuity in their coupling to cytosolic signaling partners like G proteins. Quantitative analysis across 124 mammalian GPCRs found 73% capable of activating G proteins across multiple classes with varying kinetics [30]. This points to an intricate signaling architecture adapted for specialized cellular functions. However, the structural determinants conferring selective G protein activation have been unclear.

As discussed by Masuho and coll. [30], comprehensive interaction network mapping between GPCRs and G proteins has revealed extensive contacts spanning multiple structural elements like intracellular loops and alpha helices. Residue networks differed markedly between classes of G proteins, though common hotspots like ICL2 and H8 mediate more general coupling. These findings demonstrate highly complex but selective interaction programming dictates GPCR signaling profiles. As noted in the introduction, the cannabinoid receptor CB1 represents one such promiscuous GPCR, capable of activating both its conventional coupling partner, namely inhibitory Gi/o proteins and stimulatory Gs proteins, despite a lack of structural information on such complexes. This promiscuous coupling remains poorly comprehended. Our MD-stabilized model of the CB1-Gαs complex, derived from protein–protein docking, helps address this gap by providing a first detailed structural perspective on CB1-Gαs binding. We identify a potential interface between the two proteins supported by available experimental data. Based on the novel molecular insights from our modeled structure, we propose an interaction framework that can explain the versatile capability of CB1 to couple both Gαi and Gαs proteins through distinct structural mechanisms.

A key finding from our docking and MD simulations is that the Gαs α5 helix adopts a distinct rotated conformation compared to Gi when bound to CB1, despite occupying an overlapping binding site (Figure 2). The orientation we observe shifted more towards TM5/6 and contacting ICL2 extensively, matches mutagenesis data showing that ICL2 is critical for CB1 coupling to Gs but not Gi [24]. It also agrees with the broader trend, quantified across GPCR complexes, of Gαs α5 helices interacting preferentially with TM5/6 while Gαi α5 preferentially contacts TM3/6 [27]. Remarkably, without bias, our simulations spontaneously sampled Gαs α5 conformations highly resembling experimentally resolved Gs-coupled GPCR structures (Figure 2E) through this conserved TM5/6 interaction pattern. Regarding ICL3, although it remained conformationally flexible and did not converge to a single fold, the simulations consistently showed recurrent contacts with the Gαs H4/h4–s6 region, suggesting that even in the absence of a well-defined structure, ICL3 may contribute to stabilizing the CB1–Gαs interface.

Our model suggests that oppositely charged residues on CB1 and the Gαs α5 helix interdigitate to form a zipper-like interaction network (Figure 4) that likely helps lock the helix in its distinct orientation. This electrostatic interface contrasts with the separate set of residues involved in contacts with Gi, where a key C-terminal acidic residue substitution in Gαs (D to L) appears to drive rotation of the α5 helix between complexes. These observations support the idea proposed from broader GPCR structural analyses [31,32] that the specific constellation of charged residues on the G protein α5 helix acts as an important selectivity filter between CB1 complexes with Gi versus Gs. Our findings provide an example of this mechanism, suggesting conserved polar contacts allow the same CB1 receptor residues to stabilize distinct α5 helix conformations in a selective manner through distinct interaction networks.

In line with this, Sun et al. [32] demonstrated that the mutation R > A at position 8.51 reduces cAMP accumulation in the adenosine 2B receptor, which primarily couples with Gs proteins. Similarly, Roubert et al. [33] found that the mutation R > W at position 7.56 decreases cAMP accumulation in the melanocortin 4 receptor, another receptor known to couple with Gs proteins. Both mutations involve changing positively charged residues to either negatively charged or neutral ones, resulting in decreased cAMP accumulation in vitro. These findings support the importance of these positively charged residues for Gs protein binding. On the other hand, Redfern-Nichols et al. [34] recently demonstrated that mutating the negative residue E to the positive one K at position H5.24 on the Gs protein can impact its coupling to multiple receptors structurally and functionally; further supporting our hypothesis. Collectively, these consistencies further validate our proposed structural model.

Recently, the crystal structure of the CB1 receptor in complex with the Gq protein was published [35]. This new structural information offers valuable context and provides independent support for our predicted binding model. The CB1–Gq complex adopts an overall binding mode closely resembling the one we computationally derived, which is expected given the high sequence similarity between Gαs and Gαq. This structural agreement reinforces the validity of our model. Nevertheless, the CB1–Gq structure also exhibits notable differences, particularly in the orientation and interactions of specific α5 helix residues. These variations are likely driven by differences in key residues involved in the receptor–dimer interface. Indeed, when superimposing our model with the CB1–Gq complex, several residues facing the CB1 dimer interface are not structurally conserved, as shown in Supplementary Figure S2. Such subtle discrepancies are expected given the intrinsic flexibility of GPCR intracellular loops and the context-dependent nature of GPCR–G protein engagement.

Future experimental testing of targeted CB1 mutations—particularly those involving residues predicted to interact with the Gαs α5 helix—would provide further validation of our model. In addition, the structural complex we present may guide the design of ligands or molecular probes that selectively promote Gi- or Gs-mediated signaling, helping to dissect the therapeutic relevance of each pathway. Despite these insights, our model has limitations. The absence of experimental stability data for the CB1–Gs complex restricts further refinement and highlights the need for future structural studies. Moreover, our modeling also does not consider receptor homodimers, which are increasingly recognized to play a role in signaling.

In summary, by integrating multiple computational methods, we have generated a CB1–Gs structural model that is broadly consistent with available experimental evidence. Our findings provide new mechanistic insight into the molecular determinants that enable CB1 to couple to Gs, extending our understanding of the receptor’s functional plasticity. This model offers a foundation for ongoing studies into CB1-mediated signaling and for the development of pathway-selective pharmacological agents. Understanding the G-protein coupling promiscuity of CB1 is essential for the precise modulation of downstream pathways and may ultimately facilitate the design of drugs that selectively target distinct signaling outcomes. The ability of CB1 to engage multiple G proteins underscores the complexity of (endo)cannabinoid signaling and the diverse physiological actions of cannabinoids. Continued experimental investigation will be critical to fully uncover the landscape of CB1-mediated signaling and to translate mechanistic insights into therapeutic advances.

## 4. Methods

### 4.1. Structures and Generic Numbering Systems

CB1 and G protein consensus residue interfaces were extracted from the GPCRdb (GPCRdb.org) and Gproteindb (Gproteindb.org) databases. G protein residues were identified using the generic numbering system for structurally conserved residues (CGN) [36]. The Ballesteros-Weinstein (B.W.) A numbering scheme for structurally conserved residues of class A GPCRs [37] was used to annotate CB1 residues. As initial structures for protein–protein docking we employed the CB1 structure derived from the cryo-EM structure of the human CB1-Gαi complex (PDB ID: 6N4B) [19]. For the coordinates of Gαs, we used the alpha short isoform (residues 16–394) obtained from the cryo-EM structure of the human nucleotide-free Gs heterotrimer complexed with the human calcitonin receptor-like receptor (PDB ID: 6UVA) [38]. In this structure, the helical domain (res. 47–207), not visible in the cryo-EM structure, was omitted.

### 4.2. Protein–Protein Docking Analysis

Protein–protein docking analysis was performed by the webserver ClusPro [39]. To select the best model from the 24 potential solutions generated by the ClusPro protein–protein docking analysis online server (Supplementary Table S1), we excluded any complexes in which the Gs protein was positioned within the lipid bilayer. Among the remaining models, the model with the highest number of member was chosen (as suggested in the Cluspro documentation https://cluspro.bu.edu/help.php, accessed on 20 June 2024), with weighted energy score of E: −654.84. The weighted score (−654.84) represents the overall energy of the complex, considering various energy terms, including repulsion (E_rep), attraction (E_att), electrostatic (E_elect), and Decoys as the Reference State (E_dars) energies. Each of these energy terms contributes to the total score, with specific weighting factors applied to indicate their relative influence (E = 0.40 E_rep + −0.40 E_att + 600 E_elect + 1.00 E_dars).

### 4.3. Intracellular Loop 3 Modeling

The intracellular loop 3 (ICL3), a flexible 19-residue segment critical for G protein binding, was modeled de novo within the complete CB1-Gs complex following the initial docking simulation. This approach ensured that loop conformations did not artificially influence docking calculations or binding poses. During modeling, priority was given to selecting conformations where ICL3 did not exhibit preferential interactions with either the G protein or the membrane. Conformations where the loop was overly folded or rolled up were systematically excluded. By focusing on neutral conformations, the aim was to preserve the natural flexibility of ICL3 and prevent positioning bias. This strategy minimized artificial constraints, thereby enhancing the reliability of the model and ensuring a physiologically relevant representation of the CB1-G protein complex.

### 4.4. Molecular Dynamics Protocol

The receptor was aligned toward the membrane using coordinates from the Orientations of Proteins in Membranes (OPM) database [40]. After aligning the structure, the system was embedded in a POPC-bilayer using the CHARMM-GUI webserver [41,42] and then solvated using TIP3P waters. The system charge was maintained at 0 using a 0.15 M concentration of NaCl ions. Before the relaxation step each system was submitted to a minimization procedure for 1000 steps. During the relaxation phase the system was equilibrated using the NPT ensemble with a target pressure equal to 1.01325 bar, a time-step of 2 fs and using the RATTLE algorithm for the hydrogen atoms. In this stage, the harmonic constraints were 60 progressively reduced until an elastic constant force equal to 0 kcal/mol and the temperature was increased to 300 K. All the simulations were conducted using the same non-bonded interaction parameters, with a cutoff of 9 Å and a smooth switching function applied starting with a distance of 7.5 Å. For the long-range electrostatics we used the PME methodology with a grid spacing of 1 Å. Each production phase was performed using the same parameters, with a time-step of 4 fs, and a hydrogen scaling factor of 4. The use of this time step was enabled by the application of a hydrogen mass repartitioning scheme. The parameters for the protein were obtained from the CHARMM36M forcefield [43], while lipids and solvent from the CHARMM36 forcefield [44,45,46,47].

To support the reliability of the results we performed a statistical analysis based on 12 MD runs. Each MD run was performed using the same experimental condition of the production phase, the 12 MD runs were concatenated in a whole trajectory. To build the representation spaces we concatenated the trajectories in two different ways, sampling the frames at intervals of 1 and 0.5 ns.

To quantify the structural stability of the selected region across simulations, we computed the Cα root-mean-square deviation (RMSD) relative to a common reference structure. All analyses were performed using VMD 1.9.3. For each of the 12 independent 1-μs trajectories, we analyzed the final 500 ns of simulation (corresponding to 500 frames at 1 ns/frame). The topology file and the reference structure were loaded first, with the reference serving as frame 0 for all analyses. RMSD was computed for Cα atoms of helical segments of the receptor and the α5 helix of the Gαs subunit. For each trajectory frame, the selected atoms were first aligned to the corresponding atoms in the reference structure using a least-squares fit. The RMSD between the aligned frame and the reference was then calculated, producing a per-frame RMSD time series for each replicate. Because consecutive MD frames are temporally correlated, we employed block averaging to obtain statistically meaningful estimates of structural variability. The RMSD time series for each replicate was divided into non-overlapping blocks of 50 ns (50 frames). The RMSD within each block was averaged, yielding 10 block means per trajectory. The mean and standard error of the mean (SEM) for each replicate were computed from these block averages, treating each block as independent (Table 1).

To assess reproducibility across independent trajectories, the block-averaged RMSD means from all 12 replicates were combined. The across-replicate mean and SEM were calculated by treating each replicate mean as an independent observation, thereby quantifying both temporal and replicate-to-replicate variability. The final estimate for the system was 2.28 ± 0.07 Å (mean ± SEM across 12 replicates, 50-ns blocks). This narrow range indicates that all 12 replicas converge to the same conformational ensemble, rather than sampling distinct or mutually exclusive conformations. The magnitude of the RMSD (≈2 Å) is consistent with localized, thermally driven fluctuations, not large-scale rearrangements.

### 4.5. Software for Analysis and 3D Rendering

To visualize and analyze the molecular structures and interactions, we employed the following software packages:

VMD (version 1.9.4) was utilized for structure visualization and the generation of publication-ready figures and employed for molecular dynamics simulations visualization and analysis. The ICL3 model has been obtained through molecular editing processes performed with MOE (www.chemcomp.com/).

## Figures and Tables

**Table 1:** RMSD-Based quantification of structural stability using block averaging.

Replicate	Mean RMSD [Å]	SEM [Å]
1	2.69	0.07
2	2.21	0.05
3	2.08	0.03
4	2.11	0.02
5	2.1	0.03
6	2.55	0.04
7	2.02	0.03
8	2.19	0.04
9	2.65	0.06
10	2.43	0.01
11	2.21	0.02
12	2.08	0.02

## Data Availability

Simulation data present in the study, the input files and the docking mode is available in GPCRmd (https://www.gpcrmd.org/view/2067/, accessed on 4 December 2025), any additional necessary data is available upon request.

## Supplementary Materials

The following supporting information can be downloaded at: https://www.mdpi.com/article/10.3390/ijms262411905/s1.
