# Supplementary material for: Type-1 Cannabinoid Receptor Promiscuous Coupling: Computational Insights into Receptor-G Protein Interaction Dynamics
Source: Int J Mol Sci. 2025 Dec 10;26(24):11905. doi: 10.3390/ijms262411905 (PMC12732796; doi:10.3390/ijms262411905)
Supplement: Supplementary file 1 [file ijms-26-11905-s001.zip › ijms-3974595-supplementary.pdf]

Supplementary Materials:

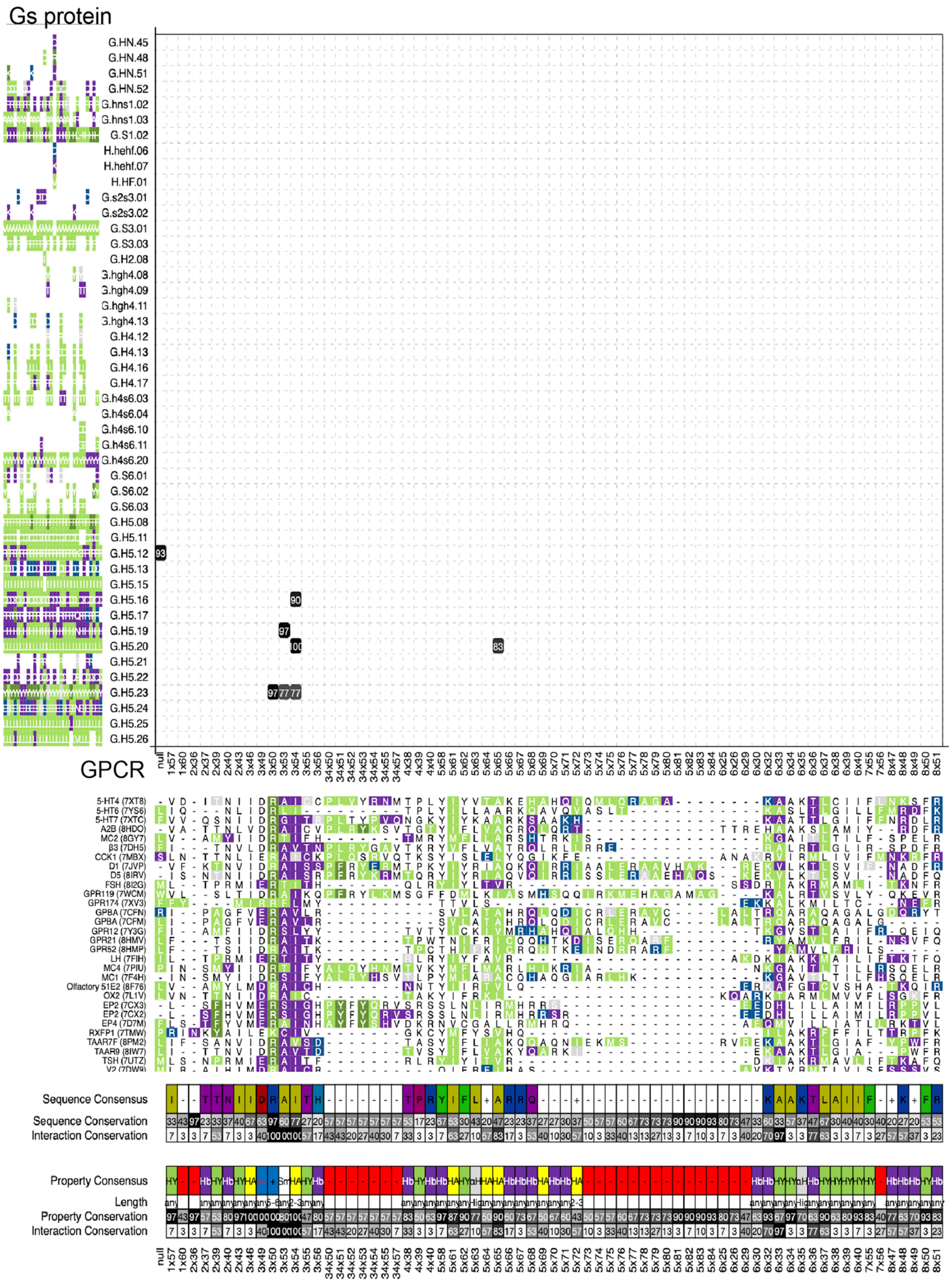

Figure S1. High-Resolution Human Class A GPCR-Gs Contact Interface Matrix: Gs-CB1 contact interface matrix derived from 30 experimentally resolved GPCR/Gs complexes representing 28 different receptors. The data were extracted from the GPCRdb database, considering only high-resolution structures of human class A GPCRs in complex with Gs. For clarity, the matrix displays only contact pairs with at least 75% conservation.

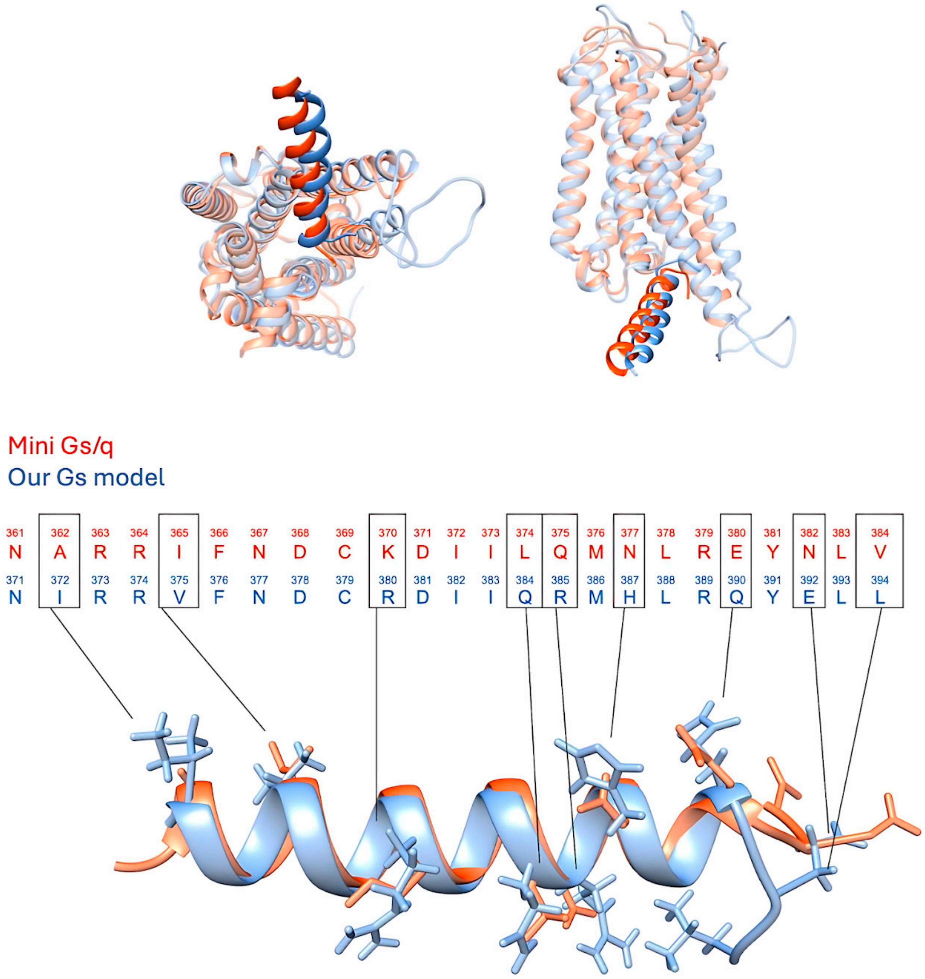

Figure S2: Mini Gq/s- Gs comparison. on the top Orientation of the 〈5 helix of the crystal structure of Gq protein (red) and Gs predicted model (blue). On the bottom 〈5 helix not conserved residues between Gq protein (red) and Gs predicted model (blue)

Cluster	Members	Representative	Weighted_Score
0	146	Center	-888.88
		Lowest_Energy	-926.16
1	129	Center	-657.79
		Lowest_Energy	-825.49
2	76	Center	-668.89
		Lowest_Energy	-826.62
3	66	Center	-735.47
		Lowest_Energy	-913.09
4 - selected cluster	61	Center	-654.84
		Lowest_Energy	-752.53
5	50	Center	-668.58
		Lowest_Energy	-722.86
6	43	Center	-680.50
		Lowest_Energy	-721.28
7	42	Center	-666.06
		Lowest_Energy	-727.98
8	41	Center	-666.58
		Lowest_Energy	-731.62
9	38	Center	-715.34
		Lowest_Energy	-746.95
10	35	Center	-679.60
		Lowest_Energy	-726.17
11	33	Center	-751.42
		Lowest_Energy	-895.49
12	30	Center	-675.42
		Lowest_Energy	-726.93
13	27	Center	-718.75
		Lowest_Energy	-794.14
14	24	Center	-654.39
		Lowest_Energy	-735.18
15	19	Center	-650.17
		Lowest_Energy	-698.98
16	19	Center	-671.07
		Lowest_Energy	-792.60
17	16	Center	-703.28
		Lowest_Energy	-742.48
18	14	Center	-664.48
		Center	-715.84
19	12	Lowest_Energy	-646.81
		Center	-680.43
20	10	Lowest_Energy	-670.92
		Center	-672.30
21	10	Lowest_Energy	-666.47
		Center	-880.11
22	10	Lowest_Energy	-646.08
		Center	-759.06
23	7	Lowest_Energy	-648.63
		Center	-678.92

Table S1: Docking scores obtained from the ClusPro protein-protein docking analysis online server. We excluded any complexes in which the Gs protein was positioned within the lipid bilayer
